# Mitigating HIV risk associated with widow cleansing and wife inheritance using combined biomedical and structural interventions in western Kenya: a mathematical modeling study

**DOI:** 10.1186/s12916-025-03906-5

**Published:** 2025-02-12

**Authors:** Duncan K. Gathungu, Viona N. Ojiambo, Mark E. Kimathi, David Kaftan, Hae-Young Kim, Daniel T. Citron, Ingrida Platais, Daniel Briedenbecker, Clark Kirkman, Samuel M. Mwalili, Anna Bershteyn

**Affiliations:** 1 Jomo Kenyatta University of Agriculture and Technology, P.O. Box 62000-00200, Nairobi, Kenya; 2 Strathmore University, Ole Sangale Road, P.O. Box 59857-00200, Nairobi, Kenya; 3 New York University Grossman School of Medicine, New York, NY USA; 4 Institute for Disease Modeling at the Bill & Melinda Gates Foundation, 500 5th Avenue North, Seattle, WA USA

**Keywords:** HIV, Modeling, Structural interventions, Empowerment, Widow, Wife inheritance

## Abstract

**Background:**

In parts of Africa, women who become widowed lose housing, bank accounts, and other property and must re-marry to avoid extreme poverty. To re-marry, some women are required to undergo widow “cleansing”—condomless sex with a man who removes “impurities” ascribed to her from her husband’s death—and are “inherited” as a wife of a brother-in-law. This study explores how HIV biomedical and structural interventions could reduce HIV-related harms associated with these practices.

**Methods:**

We adapted EMOD-HIV, an HIV agent-based network transmission model previously calibrated and validated for the Nyanza region of western Kenya. Building on the model’s pre-existing configuration of marriages, mortality, and widowhood, we added widow cleansing and wife inheritance with assumptions based on literature. Modeled HIV prevalence among inherited widows was validated to match observed data. We modeled the effect of widowed women, cleansers, and inheritors receiving biomedical HIV interventions (testing, treatment for those tested positive, and 1 year of pre-exposure prophylaxis (PrEP) initiated at cleansing for those tested negative) with or without structural interventions (female empowerment). We modeled low (30%) and high (70%) intervention uptake and reported HIV outcomes including cumulative infections over 2025–2050.

**Results:**

Modeled HIV prevalence among inherited widowed women was 59.8% (95% CI: 59.5–60.2%), comparable to observed prevalence of 64.1% (95% CI: 63.2–65.4%). Among all widowed women, biomedical interventions averted 2.0% (95% CI: 1.3–2.6%) of HIV infections with low uptake and 2.6% (95% CI: 2.0–3.2%) with high uptake. Combined biomedical and structural interventions averted 7.8% (95% CI: 7.2–8.4%) of HIV infections with low uptake and 16.1% (95% CI: 15.5–16.6%) with high uptake. Impacts were smaller for men, e.g., high-uptake structural and biomedical interventions averted 1.8% (95% CI: 1.5–2.2%) of infections among cleansers and 2.7% (95% CI: 2.4–3.0%) among inheritors.

**Conclusions:**

Widowed women are a vulnerable population with extremely high HIV prevalence. Combined biomedical and structural interventions focused on the practice of widow cleansing and wife inheritance have the potential to avert up to one-quarter of HIV infections among widowed women, and a smaller proportion among men participating in these practices.

**Supplementary Information:**

The online version contains supplementary material available at 10.1186/s12916-025-03906-5.

## Study design

In parts of Africa, women who become widowed may lose property rights, including housing, bank accounts, and valuable possessions. In order to re-marry, some women are required to undergo widow “cleansing” (i.e., sex without a condom with a man who removes “impurities” ascribed to her from her husband’s death) and are “inherited” as a wife of brother-in-law. This mathematical modeling study explores how HIV biomedical and structural interventions could reduce HIV-related harms for both widowed women and men who participate in these practices.

## Background

In parts of Africa, women who become widowed lose housing, bank accounts, and other property [1] and must re-marry to avoid extreme poverty [2]. To re-marry, some women are required to undergo “widow cleansing”—condomless sex with a man who removes “impurities” ascribed to her from her husband’s death [3]—and are “inherited” as a wife of a brother-in-law. Typically, women are expected to undergo this process within 1 year of becoming widowed, else they may risk losing the ability to re-marry and being marginalized in their communities [4].

Regions where widow cleansing and wife inheritance are practiced include parts of Kenya [5, 6], Mozambique [7], Tanzania [8], Uganda [9], and Zambia [10], all of which have been hard-hit by the HIV/AIDS pandemic. In parts of western Kenya, within a region known as Nyanza, as many as 1 in 4 adults are living with HIV—some of the highest rates observed globally. Widow cleansing and wife inheritance were outlawed in Kenya by the 2015 Act of Parliament (70–70), but the practices continue, especially in rural communities where legal enforcement is challenging [11].

Widow cleansing and wife inheritance have a bi-directional relationship with the HIV/AIDS pandemic. On one hand, HIV/AIDS has been a major driver of mortality in Africa, leading to high rates of widowhood and high HIV prevalence among widowed women, who may have acquired HIV from a husband who subsequently died of AIDS [12]. On the other hand, widow cleansing and wife inheritance may perpetuate HIV due to unprotected sexual contact between widow and the “cleanser,” between the widow and the husband who inherits her, and potentially with partners outside the marriage, given the higher probability of abusive or emotionally unfulfilling relationships resulting from marriage by inheritance [3].

In addition to cultural beliefs, other social and structural factors perpetuate widow cleansing, wife inheritance, and their association with HIV/AIDS. An important determinant of widow cleansing and wife inheritance is women’s lack of social and economic empowerment. Widowed women are more likely to undergo cleansing if they have low literacy and socioeconomic status [2]. Some do not have rights to the property previously owned by their husbands, including homes, bank accounts, and valuable possessions, but are still primary caregivers for their children [13]. For women without independent economic resources, widow cleansing and wife inheritance may be the only option to re-gain access to essential resources and avoid a life of extreme poverty.

Socioeconomic status also impacts men’s participation in these practices, but in a different manner. Men with higher socioeconomic status, levels of education, and HIV awareness may opt not to participate in these practices and to hire other men, typically of lower socioeconomic status, as substitutes [14]. These men receive compensation in exchange for cleansing or inheriting a widow. Although a product of increased HIV awareness among men, this shift toward using “professional” cleansers and inheritors concentrates HIV risk in a small population of men, further increasing HIV exposure among widowed women.

HIV stigma also contributes to the interactions of HIV and widow cleansing/wife inheritance. Widowed women may avoid disclosing HIV status for fear they may be unable to re-marry. One study found that inherited widows were less likely to disclose their HIV status compared to widows who do not undergo these practices [15]. This is particularly important because widowed women in western Kenya are three times more likely to be HIV-positive compared to married women [16] with one study recording an HIV prevalence of 64% among inherited widows [14] and another study reporting that the HIV prevalence among the widows [17] in Kenya to be as high as 44.2% and [18] reporting that the HIV prevalence among the widows to be as high as 51.8%—an HIV prevalence exceeding even the high rates seen among HIV key populations in Kenya, such as sex workers [19, 20] and men who have sex with men [21, 22].

Multiple interventions may help to address these epidemiological and social determinants of HIV risk among widowed women, including biomedical interventions to specifically address HIV-related harms, and structural interventions to address underlying determinants of these practices. Biomedical interventions, including HIV testing, treatment, and prophylaxis, could help to address HIV acquisition and transmission risk in the context of these practices. Viral load suppression through HIV treatment has been shown to both prevent HIV-related illness [23] and prevent HIV transmission to sexual partners [24], while chemoprophylaxis has been found effective at reducing the risk of HIV acquisition from sexual partners [25, 26]. Structural interventions, which help to address underlying social, political, and economic determinants of health, may be used to reduce women’s exposure to these practices. For example, legal reforms to enable widowed women to retain property rights after a husband’s death [1], and associated services such as advancement of gender equity in banking coverage [27], may reduce the economic need to undergo widow cleansing and wife inheritance [8, 28]. Economic uplift of both women and men with low levels of income and education may reduce participation, including for “professional” cleansers and inheritors [29, 30]. Education, sensitization programs on property, land and inheritance rights, and legal empowerment to take action against gender-based violence, including forced sex in the context of cultural practices, can further reduce HIV and a multitude of other harms from these practices [7].

The aim of this study was to estimate the potential impact of biomedical interventions alone and in combination with structural interventions for women and men participating in widow cleansing and wife inheritance in Kenya. We leveraged a previously calibrated and validated network-based transmission model of HIV in the Nyanza region of Kenya, together with information about widow cleansing and wife inheritance obtained from literature, to simulate this process and explore how interventions could reduce risk at the intersection of these practices and the HIV/AIDS pandemic.

## Methods

### Model description

This study leveraged an agent-based HIV network transmission model, EMOD-HIV, a component of the open-source modeling software EMOD that includes several other diseases (malaria, tuberculosis, typhoid, and others) [31]. We used EMOD version 2.2, which is available online [32] with documentation on the model’s website [33] and in literature [34].

Briefly, EMOD-HIV includes the components of population demographics, HIV disease progression and treatment, HIV transmission and prevention, and patterns of care access through a detailed continuum of care. Demographics include births resulting from age-specific fertility rates of women, mortality due to HIV, and non-HIV mortality rates stratified by age, sex, and year. Untreated HIV disease prognosis is age-dependent, and prolongation of survival with HIV treatment depends on the patient age, sex, and CD4 count at the time of treatment initiation [33]. Treatment is assigned to individuals using a modeled HIV care continuum, which includes different mechanisms of HIV testing (e.g., voluntary, antenatal, symptom-driven), linkage and retention in care, and dropout and re-initiation of care [35].

The model utilizes an age/sex-structured social network of sexual relationships. Transmission of HIV is simulated at the level of individual coital acts within relationships and depends on treatment status of an HIV-positive partner, circumcision (if male) and use of prophylaxis by an HIV-negative partner, concurrent sexually transmitted infection in either partner, and whether a condom is used. Relationships are classified into different types (marital, informal, transitory, and commercial) [36]. For each relationship type, the model was calibrated to determine the probability of being faithful, i.e., to avoid initiating any other relationships while that relationship was active. Marital partnerships, the focus of the present study, have the highest probability of being faithful of any relationship type, and were are formed at age-specific rates and matched with partners of specific ages in order to produce the age-sex patterns of marriages observed data, in this cases, the Kenya Demographic and Health Survey data for the Nyanza region [36, 37]. Prior to adaptation for the present study, the model was configured so that the death of either spouse would cause dissolution of a marriage, after which the surviving partner would be treated as an unmarried individual and be eligible to re-marry. Rates of re-marriage were assumed to be the same as those of first marriage for individuals in the same age and gender category, which were set to ensure the final age-patterns of marriage matched those of population-based surveys [38].

### Model calibration and validation for western Kenya

EMOD-HIV was previously calibrated to match data from population-based HIV surveys in the six counties of the Nyanza region in western Kenya [39, 40], and validated to predict incidence in a large, blinded prospective trial in two of the counties [41]. The counties included in the model are Homa Bay, Kisii, Kisumu, Migori, Nyamira, and Siaya. In calibration, parameters with high uncertainty—chiefly those related to sexual behavior, such as rates of causal and multiple sex partners—were tuned so that the overall modeled epidemic would match age/sex-specific prevalence from five population-based HIV surveys [39, 40]. Parameter tuning was performed using a stochastic optimization algorithm, Parallel Simultaneous Perturbation Optimization (PSPO) [42], which takes into account survey data uncertainty, in order to obtain 250 well-fitting model trajectories with unique input parameter sets. In validation, the model was fit to baseline data (demographics, HIV prevalence, risk factors such as mobility) from 16 communities of approximately 10,000 residents each in Homa Bay and Migori counties of western Kenya, which were enrolled in a prospective randomized controlled trial of treatment as prevention with HIV incidence as its primary outcome. A prediction of community-level 3-year cumulative HIV incidence was deposited to a preprint archive while the trial was blinded, and compared to the trial outcome after unblinding [41].

### Model adaptation for widow cleansing and wife inheritance

**Fig. 1:**
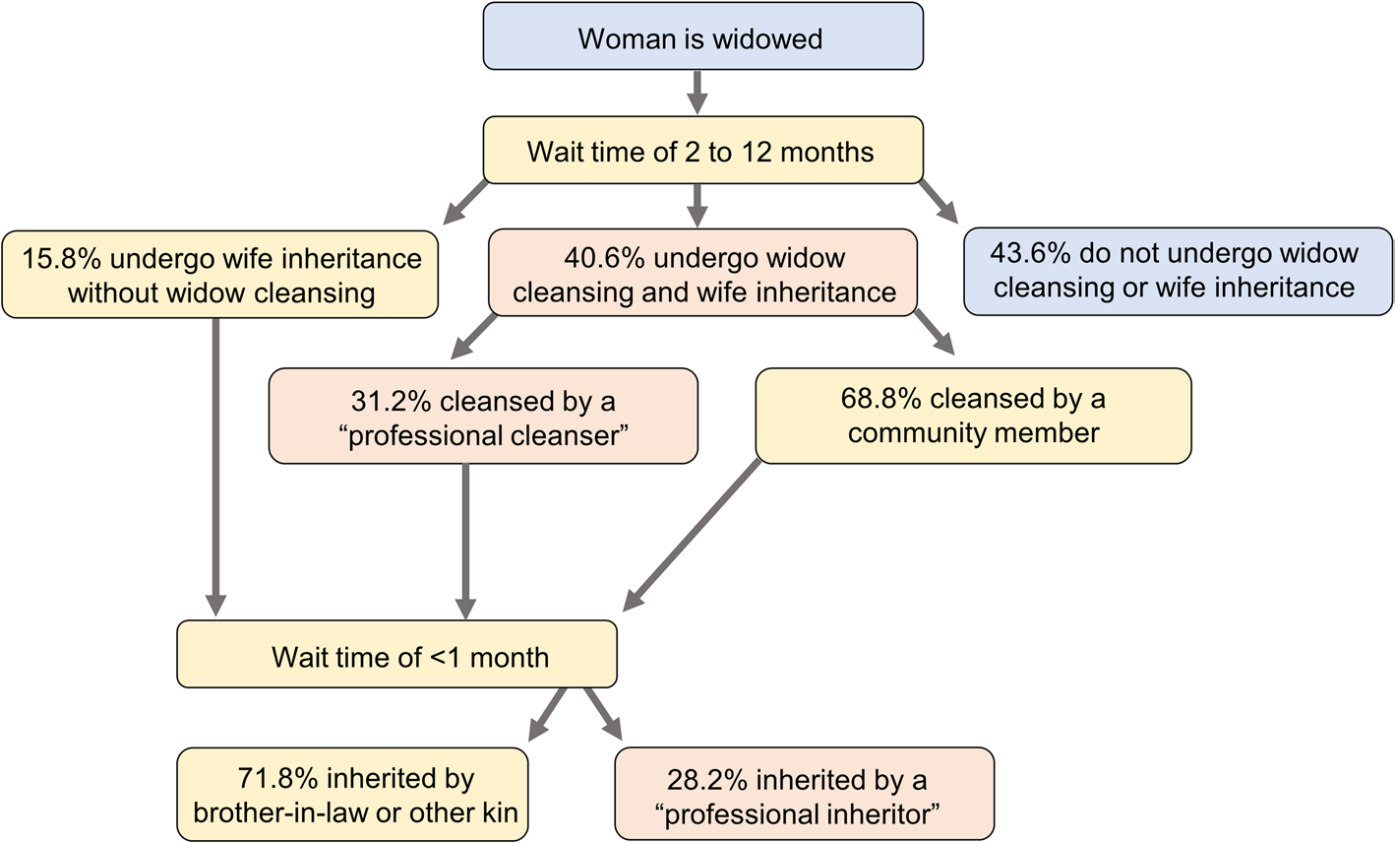
Diagram of chronological events in the process of widow cleansing and wife inheritance. All proportions were derived from Agot et al. [14]

We adapted the western Kenya EMOD-HIV model to simulate the process of widow cleansing and wife inheritance (Fig. 1). We configured the process to be triggered by the event of a death of a married male in the simulation, which included non-HIV deaths from the model’s demographics component and HIV deaths from its HIV disease progression component. Thus, the frequency with which widow cleansing and wife inheritance would occur in the model was not a model input, but depended on the rate of mortality among married men. Widow cleansing and wife inheritance used a separate mechanism from the normal re-marriage process described above. After becoming widowed, each woman was assigned a wait time. At the end of the wait time, the woman was matched with a cleanser for one condomless coital act, and subsequently matched with an inheritor to initiate a marriage. Cleansers and inheritors were categorized into “professional” and “non-professional,” ensuring that the model structure includes a smaller population of professionals who conduct a disproportionately large number of widow cleansing and wife inheritance activities. Both professional and nonprofessional inheritors were considered eligible to inherit wives even if they were already married, in which case the inheritance would lead to polygamy.

### Model assumptions

We reviewed literature to inform model assumptions regarding the process of widow cleansing and wife inheritance (Table 1). Widowed women were assigned a 41% and 56% probability of initiating the widow cleansing and wife inheritance process, respectively [14]. Forty-four percent of widows would not engage in either practice, and would re-marry at age-dependent rates as described above for the original model. Women designated for cleansing would wait between 2 and 12 months (uniformly distributed, for a mean of 7 months) from the husband’s death until the next stage of the process, widow cleansing. This reflects the expectation that women must be inherited within 1 year of becoming widowed [4]. After the wait time, a cleanser is selected. The probability of a given woman being assigned a professional cleanser (0.2% of all men) was 31%, while the probability of being assigned non-professional cleanser (30% of all men) was 69% [14]. The probability of a given woman being assigned a professional inheritor (0.2% of all men) was 28%, while the probability of being assigned a non-professional inheritor (30% of all men) was 72% [14]. For inherited marriages, the assumption that marriages reduce the initiation of additional partnerships was made to account for the higher likelihood of outside partners for arranged marriages [3] and polygamous marriages [43, 44].

**Table 1:** Model parameters for widow cleansing and wife inheritance

Parameter	Value	Source
**(i) Values used as model inputs**		
Proportion of widowed women who undergo wife inheritance	56.4%	[14]
Proportion of widowed women who undergo widow cleansing	40.6%	[14]
Proportion of all men available to perform professional (paid) cleansing	0.2%	Assumed
Proportion of all men available to perform non-professional cleansing	30%	Assumed
Proportion of cleansing performed by a professional cleanser	31%	[14]
Proportion of cleansing performed by a non-professional cleanser	69%	[14]
Proportion of all men available to be a professional (paid) inheritor	0.2%	Assumed
Proportion of all men who are available to be a non-professional inheritor	30%	Assumed
Proportion of inheritance performed by professional (paid) inheritors	28%	[14]
Proportion of inheritance performed by non-professional inheritors	72%	[14]
Delay time from husband’s death until widow cleansing occurs	2–12 months^a^	[4]
Delay time from widow cleansing until wife inheritance	< 1 month^b^	[4]
**(ii) Data used for model validation**^c^		
HIV prevalence among inherited widows, 2003–2007^d^	64.1% (95% CI: 63.2–65.4%)	[14]
HIV prevalence among women ages 15–49, 2003	18.3% (95% CI: 13.3–23.9%)	[45]
HIV prevalence among men ages 15–49, 2003	11.6% (95% CI: 6.8–17.4%)	[45]
HIV prevalence among women ages 15–49, 2007	17.6% (95% CI: 14.2–21.3%)	[46]
HIV prevalence among men ages 15–49, 2007	11.4% (95% CI: 8.5–14.6%)	[46]
HIV prevalence among women ages 15–49, 2008	16.0% (95% CI: 12.0–20.5%)	[47]
HIV prevalence among men ages 15–49, 2008	11.4% (95% CI: 7.9–15.5%)	[47]
HIV prevalence among women ages 15–49, 2012	17.6% (95% CI: 13.1–22.6%)	[48]
HIV prevalence among men ages 15–49, 2012	13.4% (95% CI: 9.3–18.1%)	[48]
HIV prevalence among women ages 15–49, 2018	8.3% (95% CI: 6.7–10.0%)	[49]
HIV prevalence among men ages 15–49, 2018	16.7% (95% CI: 14.5–19.0%)	[49]

^a^In the model, the delay time is assigned as a value randomly selected from a uniform distribution of 2 and 12 months, for a mean delay of 7 months

^b^Typical delay times are 1–2 weeks. Because the model timestep was 1 month, this delay was not modeled

^c^Additional HIV prevalence data are provided in Additional file 1: Table 1

^d^These data were not used as input. Instead, the data were used to validate that modeling the process produced correct HIV prevalence after the other listed parameters were added to the model

### Modeled interventions

Interventions to reduce HIV risk associated with widow cleansing and wife inheritance have been called for [3, 4, 7], but insufficiently studied. We configured scenarios broadly representing biomedical interventions for HIV risk reduction, with and without the addition of structural interventions. Biomedical interventions included universal testing and treatment and HIV prophylaxis. We assumed all widows, cleansers, and inheritors would be tested and immediately initiated on HIV treatment if positive. For those testing HIV-negative, we further assumed either low uptake of HIV pre-exposure prophylaxis (PrEP) resulting in a coverage of 30%, or high uptake resulting in a coverage of 70%. For inherited widows and their inheritors, PrEP was assumed to continue for 1 year after inheritance occurs. PrEP was assumed to have an efficacy of 95% if taken up. In two additional model scenarios, we added to the biomedical prevention package structural interventions to empower women to avoid exposure to widow cleansing and wife inheritance. Such a package may include economic empowerment to not rely on inheritance for poverty avoidance, legal empowerment to ensure practices that are outlawed are not practiced in their communities, and community sensitization regarding the importance of upholding women’s rights and agency. Uptake of this intervention was also assumed to reach either 30% or 70% of widowed women and reduce participation by this proportion. When combined with biomedical interventions, e.g., 30% of women would cease participation, while 30% of remaining women would receive biomedical interventions including PrEP. All interventions were applied over the years 2025–2050.

### Reported outcomes

We reported cumulative HIV infections, cumulative HIV deaths, and HIV prevalence over the period 2025–2050 for the sub-populations that are the focus of the present analysis: widowed women who are and are not exposed to widow cleansing and wife inheritance; men who perform widow cleansing; and men who inherit widowed women as wives. Reported results are averages of 250 simulation runs, with 95% confidence intervals obtained by bootstrapping individual simulation results using the Python package scipy.stats.bootstrap.

## Results

**Fig. 2:**
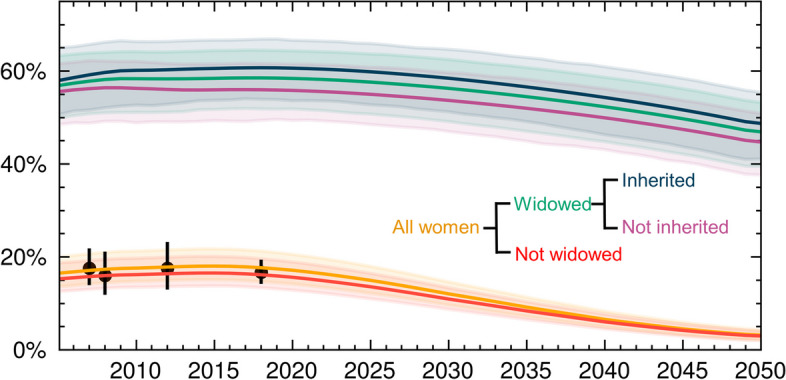
HIV prevalence among all women, widowed vs. not widowed women, and inherited vs. not inherited widowed women in western Kenya. HIV prevalence among women ages 15–49 (yellow line) is shown in comparison to observed HIV prevalence among women in the same age group from five population-based surveys (black datapoints). Model results are then subdivided by whether or not women are widowed (green vs. red lines) and whether or not widowed women are inherited (blue vs. purple lines). Shaded areas represent 95% confidence intervals

After the incorporation of widow cleansing and wife inheritance into the model, modeled women’s HIV prevalence (Fig. 2, yellow line) concurred with prevalence data from five population-based HIV surveys (Fig. 2, black datapoints; Table 1; Additional file 1: Table 1). Similarly, modeled men’s HIV prevalence concurred with data for men from the same surveys (Additional file 1: Figs. 1 and 2).

The high HIV prevalence among widows produced by the model was comparable to observed HIV prevalence. Agot et al. [14] estimated HIV prevalence among inherited widowed women in Bondo District, part of Siaya County in the Nyanza region, at 64.1% (95% CI: 63.2–65.4%). Modeled HIV prevalence among inherited widowed in Siaya in 2005, the approximate time of Agot et al.’s study, was 68.3% (95% CI 67.8–68.8). Siaya County has higher HIV prevalence than the Nyanza region overall (Additional file 1: Table 1), with the second-highest HIV prevalence among women across the six counties in this region. Accordingly, HIV prevalence among inherited widowed women was slightly lower in Nyanza overall compared to in Siaya Country: 58.0% (95% CI 57.5–58.4%) in 2005, the year of Agot et al.’s study, and 59.8% (95% CI: 59.5–60.2%) in 2025, the year when modeled interventions were initiated.

**Fig. 3:**
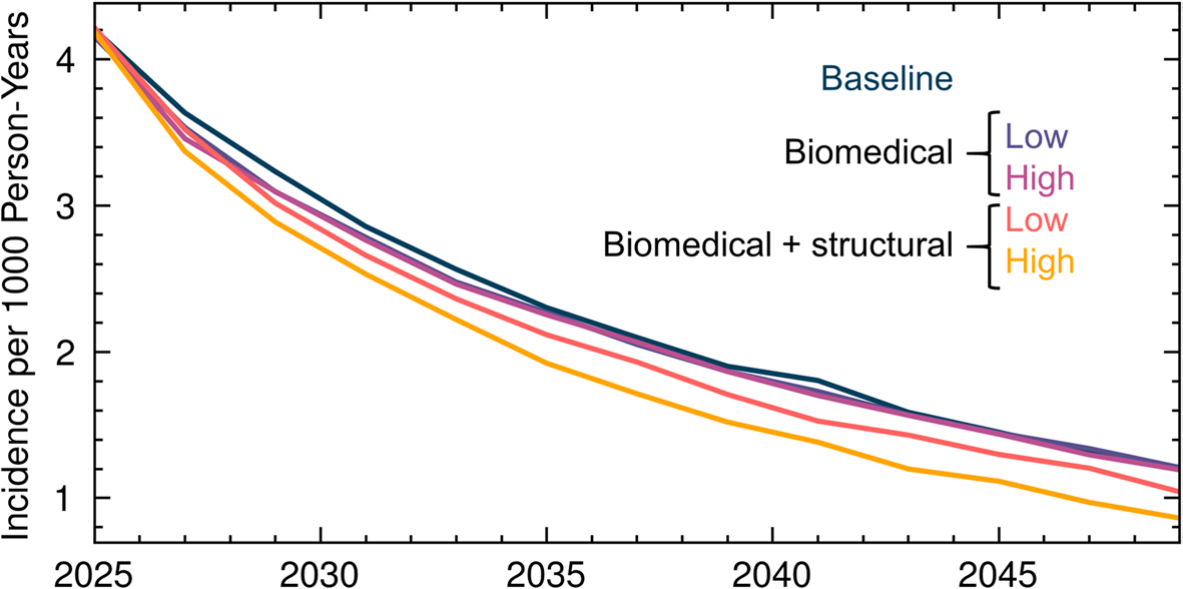
HIV incidence among widowed women with biomedical interventions alone and in combination with structural interventions, as compared to a baseline of no interventions. Biomedical interventions included universal HIV testing for widowed women, cleansers, and inheritors, with HIV treatment initiation for those tested positive. For those tested HIV-negative, biomedical interventions included either low (30%) or high (70%) uptake of HIV pre-exposure prophylaxis (PrEP) with 95% efficacy. For inherited widows and their inheritors, PrEP was assumed to continue for 1 year after the inheritance occurs. Structural interventions were assumed to reduce the exposure of widowed women to widow cleansing and wife inheritance by 30% or 70% (low vs. high uptake)

HIV prevalence among widowed women (Fig. 2, green line) was more than triple that of non-widowed women (Fig. 3, orange line). Exposure to widow cleansing and wife inheritance further increased HIV prevalence among widowed women (Fig. 2, purple vs. blue lines). HIV prevalence among widowed women who have been inherited grew over the 2000s and 2010s, while HIV prevalence among widowed women not in inherited marriages fell over this period. As a result, over the ensuing decades, HIV prevalence among women in inherited marriages exceeded prevalence in widowed women who were not inherited by an absolute difference of approximately 5%.

Among men, participation in widow cleansing and wife inheritance also profoundly impacted HIV prevalence, particularly among men who acted as professional cleansers and inheritors. HIV prevalence among non-professional cleansers was only slightly higher than among the general population of men (Additional file 1: Fig. 1, purple curve), but prevalence was substantially higher among professional cleansers (Additional file 1: Fig. 1, blue curve), exceeding that of non-professional cleansers by an absolute difference of nearly 2% by the mid-2010s. Similarly, HIV prevalence among non-professional inheritors was only slightly higher than among the general population of men (Additional file 1: Fig. 2, purple curve), but prevalence was substantially higher among professional inheritors: up to four-fold higher than that of non-professional inheritors at its peak in the early 2010s (Additional file 1: Fig. 1, blue curve).

HIV incidence among widowed women in 2025, the year when interventions were simulated, was high at 4 infections per 1000 person-years of exposure (Fig. 3). Regardless of interventions, incidence was projected to decline through 2050, reflecting the long-term effects of population-wide progress in HIV epidemic control, e.g., high rates of voluntary medical male circumcision [50] and high rates of HIV viral load suppression [49] in the Nyanza region. Biomedical and structural interventions produced relatively modest reductions in HIV incidence in their first decade (2025–2035), but by the final decade of the simulation (2040–2050), their impacts were substantial relative to the magnitude of overall incidence among widowed women. The highest-impact intervention—combined biomedical and structural interventions with high uptake—nearly reached the HIV epidemic control benchmark of < 1 infection per 1000 person-years of exposure [51] for the population of widowed women.

**Fig. 4:**
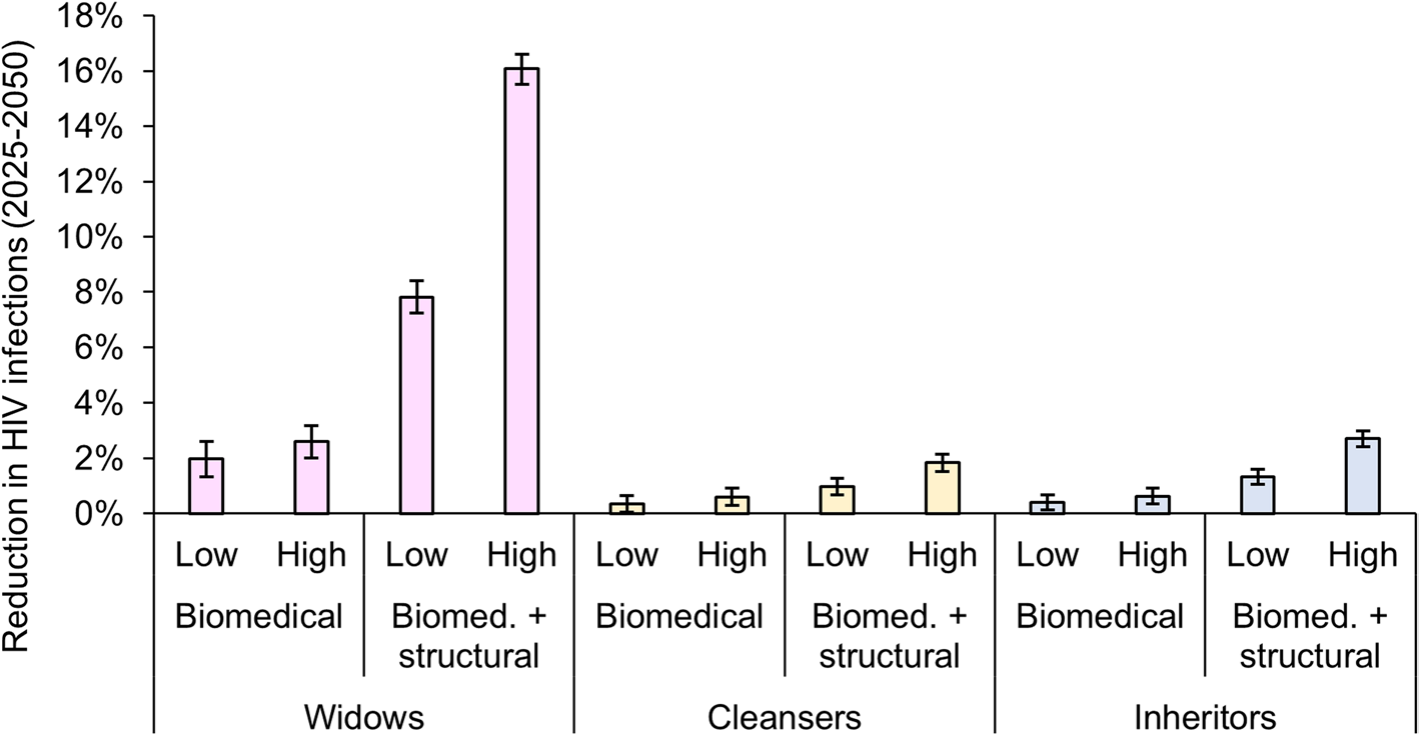
Percent reduction in HIV infections over 2025–2050 when biomedical and structural interventions are provided to women and men exposed to widow cleansing and wife inheritance. Biomedical interventions included universal HIV testing for widowed women, cleansers, and inheritors, with HIV treatment initiation for those tested positive. For those tested HIV-negative, biomedical interventions included either low (30%) or high (70%) uptake of HIV pre-exposure prophylaxis (PrEP) with 95% efficacy. For inherited widows and their inheritors, PrEP was assumed to continue for 1 year since the inheritance occurs. Structural interventions were assumed to reduce the exposure of widowed women to widow cleansing and wife inheritance by 30% or 70% (low vs. high uptake). All scenarios are compared to a scenario without biomedical or structural interventions

Over the period 2025–2050, biomedical and structural interventions had the potential to avert more than one-quarter of new HIV infections among widowed women (Fig. 4). Low uptake of biomedical interventions alone would avert only 2.0% (95% CI: 1.3–2.6%) of new HIV infections, while high uptake of biomedical interventions alone would avert 2.6% (95% CI: 2.0–3.2%) of new HIV infections. A combination of biomedical and structural interventions would have four-fold greater impact than biomedical interventions alone, averting 7.8% (95% CI: 7.2–8.4%) of new HIV infections at low uptake, or 16.1% (95% CI: 15.5–16.6%) at high uptake. Impacts on new infections were lower for men participating in widow cleansing and wife inheritance, although impacts for men were also three- to 11-fold larger when coupled with structural interventions to reduce the number of widowed women undergoing widow cleansing and wife inheritance. Impacts on HIV deaths (Additional file 1: Fig. 3) were more modest than impacts on HIV incidence, but also showed much larger impacts on widowed women compared to men participating in widow cleansing and wife inheritance.

## Discussion

This mathematical modeling study explored how HIV biomedical and structural interventions could reduce HIV-related harms associated with widow cleansing and wife inheritance. Our study confirmed that incorporating widow cleansing and wife inheritance into a mechanistic network-based transmission model reproduced the extremely high HIV prevalence—approximately 60% HIV positivity—observed among women exposed to these practices. We found that a combination of biomedical and structural interventions, the former reducing HIV acquisition rates and the latter empowering women to avoid exposure to these practices while sensitizing communities to the importance of upholding women’s rights and agency, could reduce new HIV infections among widowed women by more than 15% over the next 25 years if uptake is high. The two classes of interventions together were much more impactful than biomedical interventions alone, and impact was highly sensitive to uptake.

Prior studies have documented widespread practice of widow cleansing and wife inheritance in geographies with high HIV prevalence [3, 7–10] and speculated about their interactions with the HIV/AIDS pandemic [10–13]. This study is the first, to our knowledge, to investigate the dynamics between HIV and these practices using mathematical modeling. A strength of the study was the use of a mechanistic approach in which the widow cleansing and wife inheritance was embedded into a previously validated HIV transmission model and then validated to produce similar HIV prevalence among inherited widows compared to observed data. Our study adds insights into how these practices may perpetuate HIV transmission, adding to the considerable health and socioeconomic disparities faced by widowed women, including loss of property, sexual violence, and stigma. It suggests ways forward for combined biomedical and structural interventions to mitigate these harms.

This study has several important limitations. First, we were unable to identify data to inform some model parameters, including the proportion of men willing to conduct widow cleansing and wife inheritance when widowhood occurs in their communities, and the proportion of men willing to perform these practices for larger numbers of women in exchange for payment. Other data were available but only in limited contexts and small sample sizes, including the proportion of women who undergo widow cleansing and wife inheritance. These data are not only potentially non-representative of the specific community where data were collected, but also may not be generalizable to other communities in the region. Inaccuracies in the size of the impacted population may affect how much the modeled interventions would reduce overall population-level HIV incidence and mortality. Accordingly, we refrained from reporting impacts on the HIV epidemic as a whole until evidence accrues quantifying the proportion of individuals who participate in these practices. Larger studies are needed to generate more precise estimates and to understand how rates vary across communities and over time. Second, we modeled interventions simplistically and did not conduct detailed analyses of each intervention component (e.g., testing, treatment, PrEP, different types of female empowerment and community sensitization) nor different stages of engagement in interventions (uptake, adherence, retention). Our intention was to represent biomedical and structural interventions in a general manner, given the paucity of implementation research to shape specific interventions for these populations. As data accrue on feasible, acceptable, and effective intervention packages, simulations of the interventions should become correspondingly more detailed. Third, in our 2025–2050 forecasts, we assumed that current rates of HIV care and prevention engagement would continue. We did not incorporate potential increases in care and prevention impacts (e.g., due to new long-acting antiretroviral formulations), nor did we model potential decreases due to declining HIV/AIDS funding from international agencies and donors. Exploring these uncertainties was outside the scope of the present analysis, which was focused on widow cleansing and wife inheritance, but is an important area for future research. Finally, we reported only HIV-related outcomes. Widowed women experience numerous disparities in health and in the social determinants of health. Though not estimated in the present study, these outcomes would be important to consider as part of decision-making around biomedical and structural interventions for widowed women.

## Conclusions

Widowed women in western Kenya and other parts of Africa experience one of the highest HIV prevalence rates seen in any global population. Combined biomedical and structural interventions focused on the practice of widow cleansing and wife inheritance have the potential to avert up to one-quarter of HIV infections among widowed women over coming decades, and a smaller proportion of infections among men participating in these practices. Research is needed to design feasible, acceptable, and effective intervention packages to mitigate HIV-related and other harms associated with these practices and to address socioeconomic and health-related disparities experienced by widowed women.

## Supplementary Information

Additional file 1. Supplementary materials. Table 1. Additional HIV prevalence data from population-based surveys. Figure 1 HIV prevalence among all men, men available for widow cleansing vs. not available for widow cleansing, and men available as professional vs. non-professional widow cleansers. Figure 2 HIV prevalence among all men, men available for widow inheritance vs. not available for widow inheritance, and men available as professional vs. non-professional widow inheritors. Figure 3 Percent reduction in HIV deaths when biomedical and structural interventions are provided to women and men exposed to widow cleansing and wife inheritance in western Kenya, 2025–2050. Figure 4 Percent reduction in HIV prevalence in 2050 when biomedical and structural interventions are provided to women and men exposed to widow cleansing and wife inheritance in western Kenya. Figure 5 Percent reduction in new HIV infections when biomedical and structural interventions are provided, and sensitivity to assumed inheritance and cleansing participation rates.

## Data Availability

Source code for the mathematical model is available online. Ref [32]

## Abbreviations

AIDS: Acquired immunodeficiency syndrome
CD4: Cluster of differentiation 4
EMOD: Epidemiological MODeling software
HIV: Human immunodeficiency virus
PrEP: Pre-exposure prophylaxis
PSPO: Parallel Simultaneous Perturbation Optimization
