# Supplementary material for: Mitigating HIV risk associated with widow cleansing and wife inheritance using combined biomedical and structural interventions in western Kenya: a mathematical modeling study
Source: BMC Med. 2025 Feb 12;23:88. doi: 10.1186/s12916-025-03906-5 (PMC11823008; doi:10.1186/s12916-025-03906-5)
Supplement: Supplementary file 1 — Additional file 1. Supplementary materials. Table 1. Additional HIV prevalence data from population-based surveys. Figure 1 HIV prevalence among all men, men available for widow cleansing vs. not available for widow cleansing, and men available as professional vs. non-professional widow cleansers. Figure 2 HIV prevalence among all men, men available for widow inheritance vs. not available for widow inheritance, and men available as professional vs. non-professional widow inheritors. Figure 3 Percent reduction in HIV deaths when biomedical and structural interventions are provided to women and men exposed to widow cleansing and wife inheritance in western Kenya, 2025–2050. Figure 4 Percent reduction in HIV prevalence in 2050 when biomedical and structural interventions are provided to women and men exposed to widow cleansing and wife inheritance in western Kenya. Figure 5 Percent reduction in new HIV infections when biomedical and structural interventions are provided, and sensitivity to assumed inheritance and cleansing participation rates. [file 12916_2025_3906_MOESM1_ESM.docx]

**Supplementary materials for:**

**Mitigating HIV risk associated with widow cleansing and wife inheritance using combined biomedical and structural interventions in western Kenya: A mathematical modeling study.**

Duncan K. Gathungu^1,2*^, Viona N. Ojiambo^1*^, Mark E. Kimathi^2^, David Kaftan^3^, Hae-Young Kim^3^, Daniel T. Citron^3^, Ingrida Platais^3^, Daniel Briedenbecker^4^, Clark Kirkman IV^4^, Samuel M. Mwalili^1,2^, Anna Bershteyn^3^

^1^Jomo Kenyatta University of Agriculture and Technology, P.O. Box 62000-00200, Nairobi Kenya.

^2^Strathmore University, Ole Sangale Road, P.O. Box 59857-00200, Nairobi, Kenya.

^3^New York University Grossman School of Medicine, New York, New York, USA.

^4^Institute for Disease Modeling at the Bill & Melinda Gates Foundation, 500 5^th^ Avenue North, Seattle, Washington, USA.

* These authors contributed equally. ^§^Correspondence: Anna.Bershteyn@nyulangone.org

**Table 1**: Additional HIV prevalence data from population-based surveys.

| County | Year | Age Group | Gender | HIV Prevalence |
| --- | --- | --- | --- | --- |
| Homa Bay | 2003 | [15:50) | Male | 11.0% (95% CI: 1.8%–27.0%) |
| Kisii | 2003 | [15:50) | Male | 1.1% (95% CI: 0.0%–4.8%) |
| Kisumu | 2003 | [15:50) | Male | 16.6% (95% CI: 8.3%–27.1%) |
| Migori | 2003 | [15:50) | Male | 18.0% (95% CI: 4.2%–38.9%) |
| Nyamira | 2003 | [15:50) | Male | 0.3% (95% CI: 0.0%–1.5%) |
| Siaya | 2003 | [15:50) | Male | 18.2% (95% CI: 12.7%–24.5%) |
| Homa Bay | 2007 | [15:50) | Male | 25.1% (95% CI: 17.2%–34.0%) |
| Kisii | 2007 | [15:50) | Male | 4.4% (95% CI: 2.6%–6.7%) |
| Kisumu | 2007 | [15:50) | Male | 11.4% (95% CI: 6.5%–17.5%) |
| Migori | 2007 | [15:50) | Male | 16.9% (95% CI: 10.3%–24.6%) |
| Siaya | 2007 | [15:50) | Male | 14.4% (95% CI: 9.3%–20.5%) |
| Homa Bay | 2009 | [15:50) | Male | 17.4% (95% CI: 10.1%–26.1%) |
| Kisii | 2009 | [15:50) | Male | 3.3% (95% CI: 0.9%–7.1%) |
| Kisumu | 2009 | [15:50) | Male | 11.1% (95% CI: 5.0%–19.1%) |
| Migori | 2009 | [15:50) | Male | 19.2% (95% CI: 10.2%–30.3%) |
| Nyamira | 2009 | [15:50) | Male | 2.3% (95% CI: 0.1%–7.5%) |
| Siaya | 2009 | [15:50) | Male | 15.3% (95% CI: 11.0%–20.1%) |
| Homa Bay | 2012 | [15:50) | Male | 22.2% (95% CI: 15.6%–29.5%) |
| Kisii | 2012 | [15:50) | Male | 3.5% (95% CI: 1.1%–7.1%) |
| Kisumu | 2012 | [15:50) | Male | 19.4% (95% CI: 10.9%–29.6%) |
| Migori | 2012 | [15:50) | Male | 14.4% (95% CI: 6.9%–23.9%) |
| Nyamira | 2012 | [15:50) | Male | 4.2% (95% CI: 1.3%–8.6%) |
| Siaya | 2012 | [15:50) | Male | 26.0% (95% CI: 17.8%–35.1%) |
| Homa Bay | 2003 | [15:50) | Female | 24.6% (95% CI: 10.8%–41.8%) |
| Kisii | 2003 | [15:50) | Female | 8.5% (95% CI: 4.1%–14.4%) |
| Kisumu | 2003 | [15:50) | Female | 19.1% (95% CI: 10.5%–29.6%) |
| Migori | 2003 | [15:50) | Female | 18.6% (95% CI: 6.1%–36.0%) |
| Nyamira | 2003 | [15:50) | Female | 7.4% (95% CI: 1.2%–18.7%) |
| Siaya | 2003 | [15:50) | Female | 24.2% (95% CI: 15.1%–34.7%) |
| Homa Bay | 2007 | [15:50) | Female | 32.6% (95% CI: 28.4%–37.0%) |
| Kisii | 2007 | [15:50) | Female | 6.9% (95% CI: 4.9%–9.2%) |
| Kisumu | 2007 | [15:50) | Female | 18.5% (95% CI: 11.2%–27.0%) |
| Migori | 2007 | [15:50) | Female | 21.8% (95% CI: 14.9%–29.7%) |
| Siaya | 2007 | [15:50) | Female | 21.3% (95% CI: 17.7%–25.2%) |
| Homa Bay | 2009 | [15:50) | Female | 25.2% (95% CI: 18.3%–32.9%) |
| Kisii | 2009 | [15:50) | Female | 5.7% (95% CI: 2.0%–11.2%) |
| Kisumu | 2009 | [15:50) | Female | 18.1% (95% CI: 11.9%–25.3%) |
| Migori | 2009 | [15:50) | Female | 22.3% (95% CI: 17.8%–27.1%) |
| Nyamira | 2009 | [15:50) | Female | 5.4% (95% CI: 1.8%–10.9%) |
| Siaya | 2009 | [15:50) | Female | 19.2% (95% CI: 9.0%–32.1%) |
| Homa Bay | 2012 | [15:50) | Female | 27.9% (95% CI: 22.2%–33.9%) |
| Kisii | 2012 | [15:50) | Female | 3.7% (95% CI: 1.6%–6.6%) |
| Kisumu | 2012 | [15:50) | Female | 20.2% (95% CI: 16.5%–24.2%) |
| Migori | 2012 | [15:50) | Female | 19.2% (95% CI: 9.1%–32.1%) |
| Nyamira | 2012 | [15:50) | Female | 10.4% (95% CI: 7.3%–14.1%) |
| Siaya | 2012 | [15:50) | Female | 29.9% (95% CI: 21.6%–39.0%) |
| Homa Bay | 2018 | [15:50) | Both | 19.1% (95% CI: 14.9%–23.8%) |
| Homa Bay | 2018 | [15:50) | Female | 25.3% (95% CI: 19.5%–31.7%) |
| Homa Bay | 2018 | [15:50) | Male | 12.8% (95% CI: 9.7%–16.2%) |
| Kisii | 2018 | [15:50) | Both | 5.7% (95% CI: 2.8%–9.6%) |
| Kisii | 2018 | [15:50) | Female | 6.8% (95% CI: 3.8%–10.7%) |
| Kisii | 2018 | [15:50) | Male | 4.6% (95% CI: 1.4%–9.5%) |
| Kisumu | 2018 | [15:50) | Both | 15.6% (95% CI: 11.5%–20.1%) |
| Kisumu | 2018 | [15:50) | Female | 21.0% (95% CI: 15.0%–27.6%) |
| Kisumu | 2018 | [15:50) | Male | 9.6% (95% CI: 5.9%–14.1%) |
| Migori | 2018 | [15:50) | Both | 12.6% (95% CI: 8.7%–17.1%) |
| Migori | 2018 | [15:50) | Female | 17.6% (95% CI: 12.8%–22.9%) |
| Migori | 2018 | [15:50) | Male | 7.1% (95% CI: 3.4%–11.8%) |
| Nyamira | 2018 | [15:50) | Both | 3.5% (95% CI: 2.0%–5.3%) |
| Nyamira | 2018 | [15:50) | Female | 4.3% (95% CI: 2.2%–7.1%) |
| Nyamira | 2018 | [15:50) | Male | 2.5% (95% CI: 0.7%–5.3%) |
| Siaya | 2018 | [15:50) | Both | 14.3% (95% CI: 11.0%–17.9%) |
| Siaya | 2018 | [15:50) | Female | 19.1% (95% CI: 14.3%–24.4%) |
| Siaya | 2018 | [15:50) | Male | 9.6% (95% CI: 6.2%–13.7%) |
| All Nyanza counties | 2003 | [15:20) | Male | 0.1% (95% CI: 0.0%–0.9%) |
| All Nyanza counties | 2003 | [20:25) | Male | 5.6% (95% CI: 0.7%–15.2%) |
| All Nyanza counties | 2003 | [25:30) | Male | 24.3% (95% CI: 11.0%–40.8%) |
| All Nyanza counties | 2003 | [30:35) | Male | 18.4% (95% CI: 4.2%–40.0%) |
| All Nyanza counties | 2003 | [35:40) | Male | 20.6% (95% CI: 6.2%–40.9%) |
| All Nyanza counties | 2003 | [40:45) | Male | 25.3% (95% CI: 9.7%–45.4%) |
| All Nyanza counties | 2003 | [45:50) | Male | 16.2% (95% CI: 3.4%–36.5%) |
| All Nyanza counties | 2003 | [50:55) | Male | 21.5% (95% CI: 6.1%–43.2%) |
| All Nyanza counties | 2007 | [15:20) | Male | 1.2% (95% CI: 0.1%–3.6%) |
| All Nyanza counties | 2007 | [20:25) | Male | 2.6% (95% CI: 0.7%–5.6%) |
| All Nyanza counties | 2007 | [25:30) | Male | 19.6% (95% CI: 10.1%–31.1%) |
| All Nyanza counties | 2007 | [30:35) | Male | 25.8% (95% CI: 15.6%–37.5%) |
| All Nyanza counties | 2007 | [35:40) | Male | 23.8% (95% CI: 12.3%–37.8%) |
| All Nyanza counties | 2007 | [40:45) | Male | 20.2% (95% CI: 9.8%–33.3%) |
| All Nyanza counties | 2007 | [45:50) | Male | 21.0% (95% CI: 11.0%–33.2%) |
| All Nyanza counties | 2007 | [50:55) | Male | 18.3% (95% CI: 8.4%–31.1%) |
| All Nyanza counties | 2008 | [15:20) | Male | 1.8% (95% CI: 0.1%–6.3%) |
| All Nyanza counties | 2008 | [20:25) | Male | 5.8% (95% CI: 0.8%–15.1%) |
| All Nyanza counties | 2008 | [25:30) | Male | 24.5% (95% CI: 14.2%–36.6%) |
| All Nyanza counties | 2008 | [30:35) | Male | 15.3% (95% CI: 5.6%–28.7%) |
| All Nyanza counties | 2008 | [35:40) | Male | 22.7% (95% CI: 8.9%–40.7%) |
| All Nyanza counties | 2008 | [40:45) | Male | 25.0% (95% CI: 8.2%–47.4%) |
| All Nyanza counties | 2008 | [45:50) | Male | 13.3% (95% CI: 1.2%–36.5%) |
| All Nyanza counties | 2008 | [50:55) | Male | 15.9% (95% CI: 4.0%–33.9%) |
| All Nyanza counties | 2012 | [15:20) | Male | 1.5% (95% CI: 0.0%–5.7%) |
| All Nyanza counties | 2012 | [20:25) | Male | 2.9% (95% CI: 0.1%–10.2%) |
| All Nyanza counties | 2012 | [25:30) | Male | 21.1% (95% CI: 6.7%–40.9%) |
| All Nyanza counties | 2012 | [30:35) | Male | 23.9% (95% CI: 13.3%–36.5%) |
| All Nyanza counties | 2012 | [35:40) | Male | 19.5% (95% CI: 8.0%–34.7%) |
| All Nyanza counties | 2012 | [40:45) | Male | 31.3% (95% CI: 16.5%–48.4%) |
| All Nyanza counties | 2012 | [45:50) | Male | 16.2% (95% CI: 4.2%–34.3%) |
| All Nyanza counties | 2012 | [50:55) | Male | 20.7% (95% CI: 9.9%–34.2%) |
| All Nyanza counties | 2003 | [15:20) | Female | 4.6% (95% CI: 1.3%–9.7%) |
| All Nyanza counties | 2003 | [20:25) | Female | 30.0% (95% CI: 19.4%–41.7%) |
| All Nyanza counties | 2003 | [25:30) | Female | 23.0% (95% CI: 11.3%–37.4%) |
| All Nyanza counties | 2003 | [30:35) | Female | 16.3% (95% CI: 6.0%–30.5%) |
| All Nyanza counties | 2003 | [35:40) | Female | 18.4% (95% CI: 6.5%–34.7%) |
| All Nyanza counties | 2003 | [40:45) | Female | 35.0% (95% CI: 15.9%–57.1%) |
| All Nyanza counties | 2003 | [45:50) | Female | 16.5% (95% CI: 1.3%–45.2%) |
| All Nyanza counties | 2007 | [15:20) | Female | 7.7% (95% CI: 3.6%–13.3%) |
| All Nyanza counties | 2007 | [20:25) | Female | 20.6% (95% CI: 14.6%–27.3%) |
| All Nyanza counties | 2007 | [25:30) | Female | 24.5% (95% CI: 17.5%–32.3%) |
| All Nyanza counties | 2007 | [30:35) | Female | 25.8% (95% CI: 16.1%–36.8%) |
| All Nyanza counties | 2007 | [35:40) | Female | 22.3% (95% CI: 14.9%–30.7%) |
| All Nyanza counties | 2007 | [40:45) | Female | 18.0% (95% CI: 9.4%–28.6%) |
| All Nyanza counties | 2007 | [45:50) | Female | 12.9% (95% CI: 6.3%–21.4%) |
| All Nyanza counties | 2008 | [15:20) | Female | 10.8% (95% CI: 5.4%–17.8%) |
| All Nyanza counties | 2008 | [20:25) | Female | 12.0% (95% CI: 6.9%–18.2%) |
| All Nyanza counties | 2008 | [25:30) | Female | 22.3% (95% CI: 11.6%–35.2%) |
| All Nyanza counties | 2008 | [30:35) | Female | 25.9% (95% CI: 12.2%–42.7%) |
| All Nyanza counties | 2008 | [35:40) | Female | 22.6% (95% CI: 11.9%–35.6%) |
| All Nyanza counties | 2008 | [40:45) | Female | 9.3% (95% CI: 2.0%–21.3%) |
| All Nyanza counties | 2008 | [45:50) | Female | 17.2% (95% CI: 5.7%–33.2%) |
| All Nyanza counties | 2012 | [15:20) | Female | 4.9% (95% CI: 1.6%–9.7%) |
| All Nyanza counties | 2012 | [20:25) | Female | 14.1% (95% CI: 7.8%–21.9%) |
| All Nyanza counties | 2012 | [25:30) | Female | 24.5% (95% CI: 15.1%–35.4%) |
| All Nyanza counties | 2012 | [30:35) | Female | 20.5% (95% CI: 11.2%–31.7%) |
| All Nyanza counties | 2012 | [35:40) | Female | 28.1% (95% CI: 16.2%–41.8%) |
| All Nyanza counties | 2012 | [40:45) | Female | 16.9% (95% CI: 7.3%–29.5%) |
| All Nyanza counties | 2012 | [45:50) | Female | 22.9% (95% CI: 11.4%–36.9%) |
| All Nyanza counties | 2018 | [15:20) | Both | 1.6% (95% CI: 0.8%–2.6%) |
| All Nyanza counties | 2018 | [15:20) | Female | 2.9% (95% CI: 1.5%–4.9%) |
| All Nyanza counties | 2018 | [15:20) | Male | 0.2% (95% CI: 0.0%–0.8%) |
| All Nyanza counties | 2018 | [20:25) | Both | 5.9% (95% CI: 3.8%–8.4%) |
| All Nyanza counties | 2018 | [20:25) | Female | 9.1% (95% CI: 5.8%–13.1%) |
| All Nyanza counties | 2018 | [20:25) | Male | 2.2% (95% CI: 0.4%–5.5%) |
| All Nyanza counties | 2018 | [25:30) | Both | 15.5% (95% CI: 11.8%–19.6%) |
| All Nyanza counties | 2018 | [25:30) | Female | 23.3% (95% CI: 18.6%–28.5%) |
| All Nyanza counties | 2018 | [25:30) | Male | 7.0% (95% CI: 3.2%–12.3%) |
| All Nyanza counties | 2018 | [30:35) | Both | 18.8% (95% CI: 15.0%–23.0%) |
| All Nyanza counties | 2018 | [30:35) | Female | 27.0% (95% CI: 22.1%–32.1%) |
| All Nyanza counties | 2018 | [30:35) | Male | 9.1% (95% CI: 5.1%–14.0%) |
| All Nyanza counties | 2018 | [35:40) | Both | 21.8% (95% CI: 17.5%–26.5%) |
| All Nyanza counties | 2018 | [35:40) | Female | 27.1% (95% CI: 19.7%–35.1%) |
| All Nyanza counties | 2018 | [35:40) | Male | 16.7% (95% CI: 11.8%–22.4%) |
| All Nyanza counties | 2018 | [40:45) | Both | 24.3% (95% CI: 19.1%–29.9%) |
| All Nyanza counties | 2018 | [40:45) | Female | 27.7% (95% CI: 21.8%–33.9%) |
| All Nyanza counties | 2018 | [40:45) | Male | 20.8% (95% CI: 13.9%–28.6%) |
| All Nyanza counties | 2018 | [45:50) | Both | 23.7% (95% CI: 18.0%–29.8%) |
| All Nyanza counties | 2018 | [45:50) | Female | 19.9% (95% CI: 13.8%–26.8%) |
| All Nyanza counties | 2018 | [45:50) | Male | 28.0% (95% CI: 18.3%–38.8%) |
| All Nyanza counties | 2018 | [50:55) | Both | 26.2% (95% CI: 19.5%–33.6%) |
| All Nyanza counties | 2018 | [50:55) | Female | 29.1% (95% CI: 18.5%–40.9%) |
| All Nyanza counties | 2018 | [50:55) | Male | 23.1% (95% CI: 14.6%–32.9%) |
| All Nyanza counties | 2018 | [55:60) | Both | 12.0% (95% CI: 7.3%–17.8%) |
| All Nyanza counties | 2018 | [55:60) | Female | 13.7% (95% CI: 7.6%–21.1%) |
| All Nyanza counties | 2018 | [55:60) | Male | 10.4% (95% CI: 4.9%–17.7%) |
| All Nyanza counties | 2018 | [60:65) | Both | 14.1% (95% CI: 8.7%–20.7%) |
| All Nyanza counties | 2018 | [60:65) | Female | 14.7% (95% CI: 7.6%–23.7%) |
| All Nyanza counties | 2018 | [60:65) | Male | 13.4% (95% CI: 5.6%–23.9%) |

**Supplementary Figures**


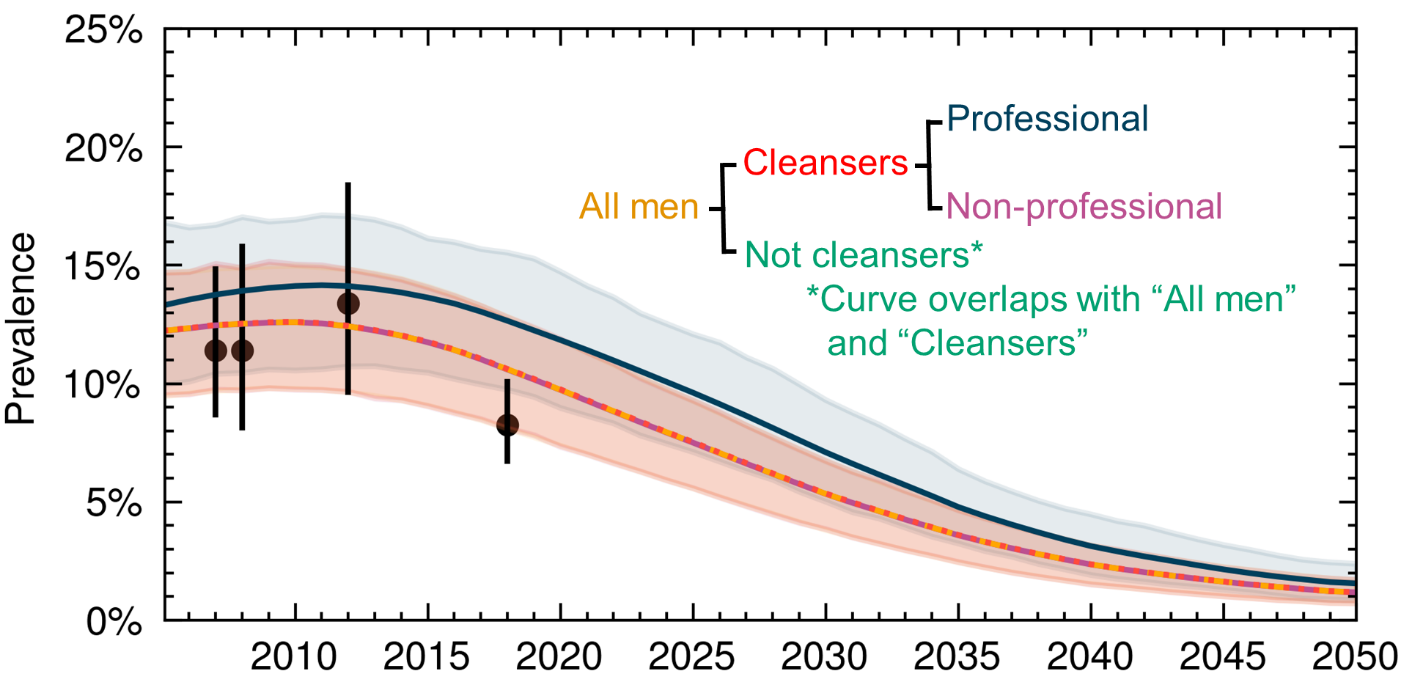


**Figure 1:** **HIV prevalence among all men, men available for widow cleansing vs. not available for widow cleansing, and men available as professional vs. non-professional widow cleansers.** HIV prevalence among men ages 15-49 (yellow line) is shown in comparison to observed HIV prevalence among men in this age group from five population-based surveys (black datapoints). Model results are then subdivided by whether or not men are available to perform widow cleansing (green vs. red lines) and whether or not widow cleansers are professional or non-professional (blue vs. purple lines). Shaded areas represent 95% confidence intervals.


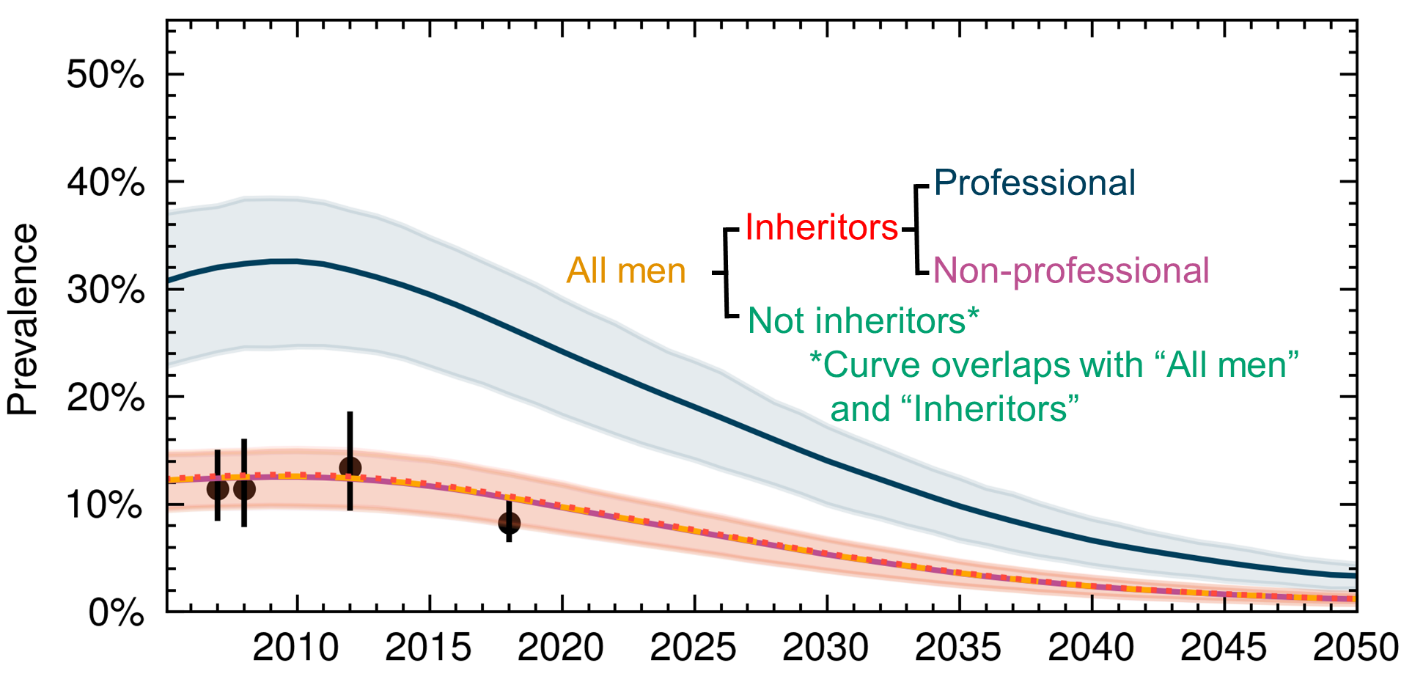


**Figure 2:** **HIV prevalence among all men, men available for widow inheritance vs. not available for widow inheritance, and men available as professional vs. non-professional widow inheritors.** HIV prevalence among men ages 15-49 (yellow line) is shown in comparison to observed HIV prevalence among men in this age group from five population-based surveys (black datapoints). Model results are then subdivided by whether or not men are available to perform widow inheritance (green vs. red lines) and whether or not widow cleansers are professional or non-professional (blue vs. purple lines). Shaded areas represent 95% confidence intervals.


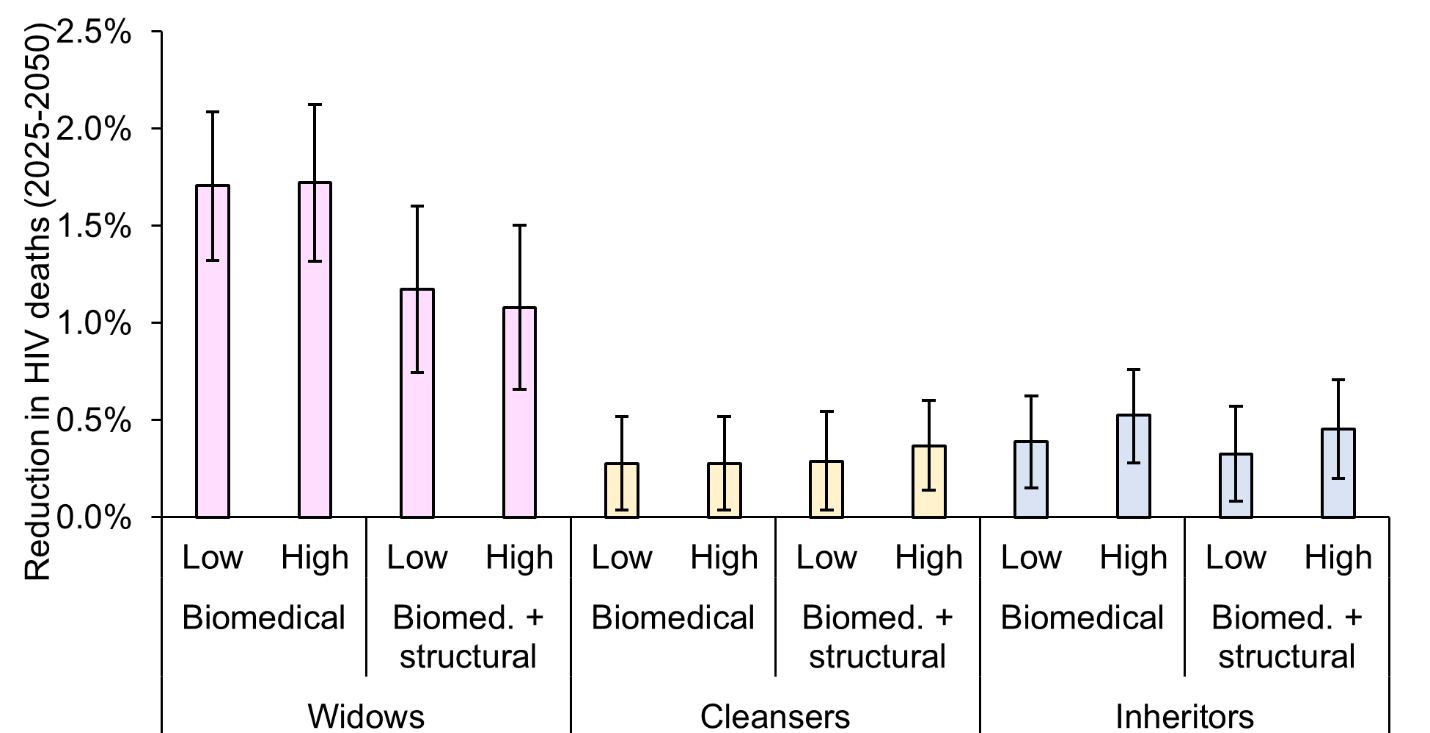


**Figure 3: Percent reduction in HIV deaths when biomedical and structural interventions are provided to women and men exposed to widow cleansing and wife inheritance in western Kenya, 2025-2050.** Biomedical interventions included universal HIV testing for widowed women, cleansers, and inheritors, with HIV treatment initiation for those tested positive. For those tested HIV-negative, biomedical interventions included either low (30%) or high (70%) uptake of HIV pre-exposure prophylaxis (PrEP) with 95% efficacy. For inherited widows and their inheritors, PrEP was assumed to continue for one year after the inheritance occurs. Structural interventions were assumed to reduce the exposure of widowed women to widow cleansing and wife inheritance by 30% or 70% (low vs. high uptake). All scenarios are compared to a scenario without biomedical or structural interventions.


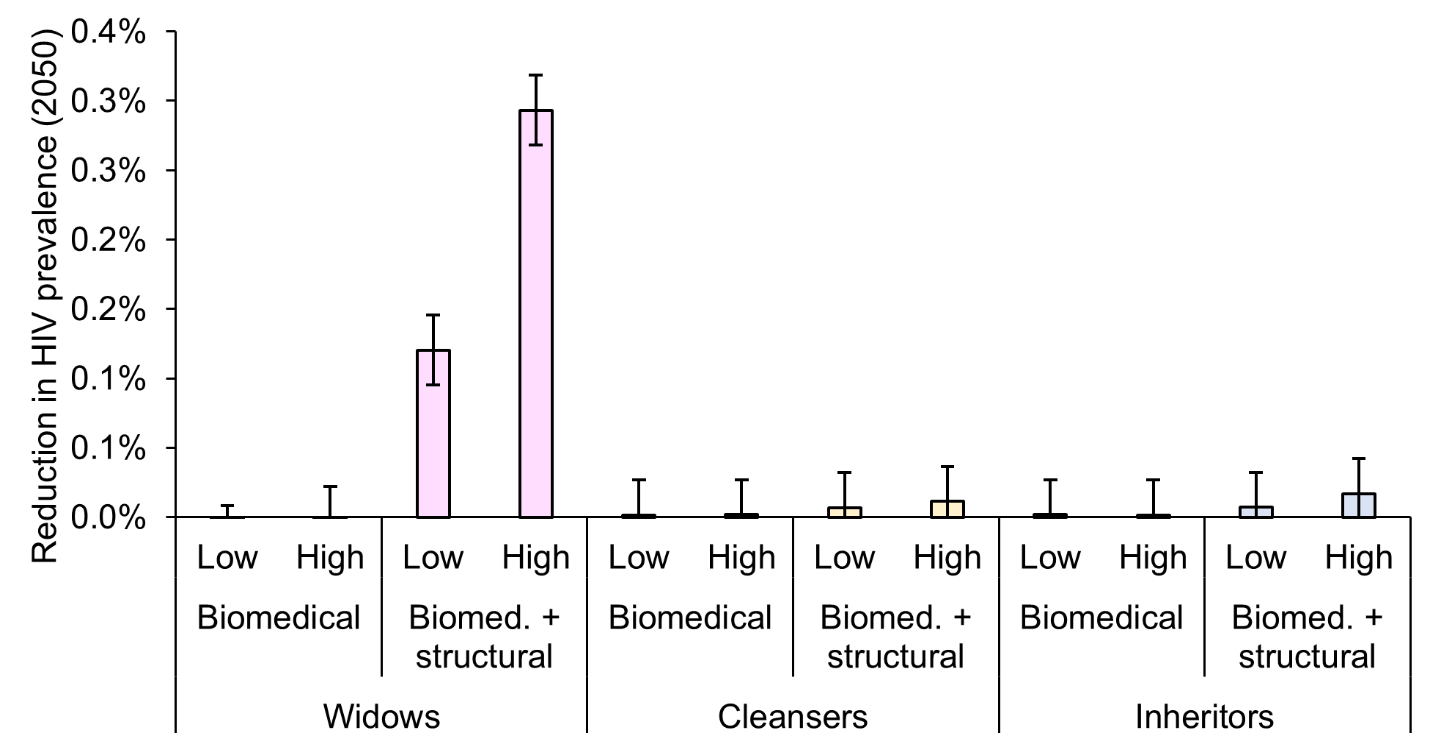


**Figure 4: Percent reduction in HIV prevalence in 2050 when biomedical and structural interventions are provided to women and men exposed to widow cleansing and wife inheritance in western Kenya.** Biomedical interventions included universal HIV testing for widowed women, cleansers, and inheritors, with HIV treatment initiation for those tested positive. For those tested HIV-negative, biomedical interventions included either low (30%) or high (70%) uptake of HIV pre-exposure prophylaxis (PrEP) with 95% efficacy. For inherited widows and their inheritors, PrEP was assumed to continue for one year after the inheritance occurs. Structural interventions were assumed to reduce the exposure of widowed women to widow cleansing and wife inheritance by 30% or 70% (low vs. high uptake). All scenarios are compared to a scenario without biomedical or structural interventions.


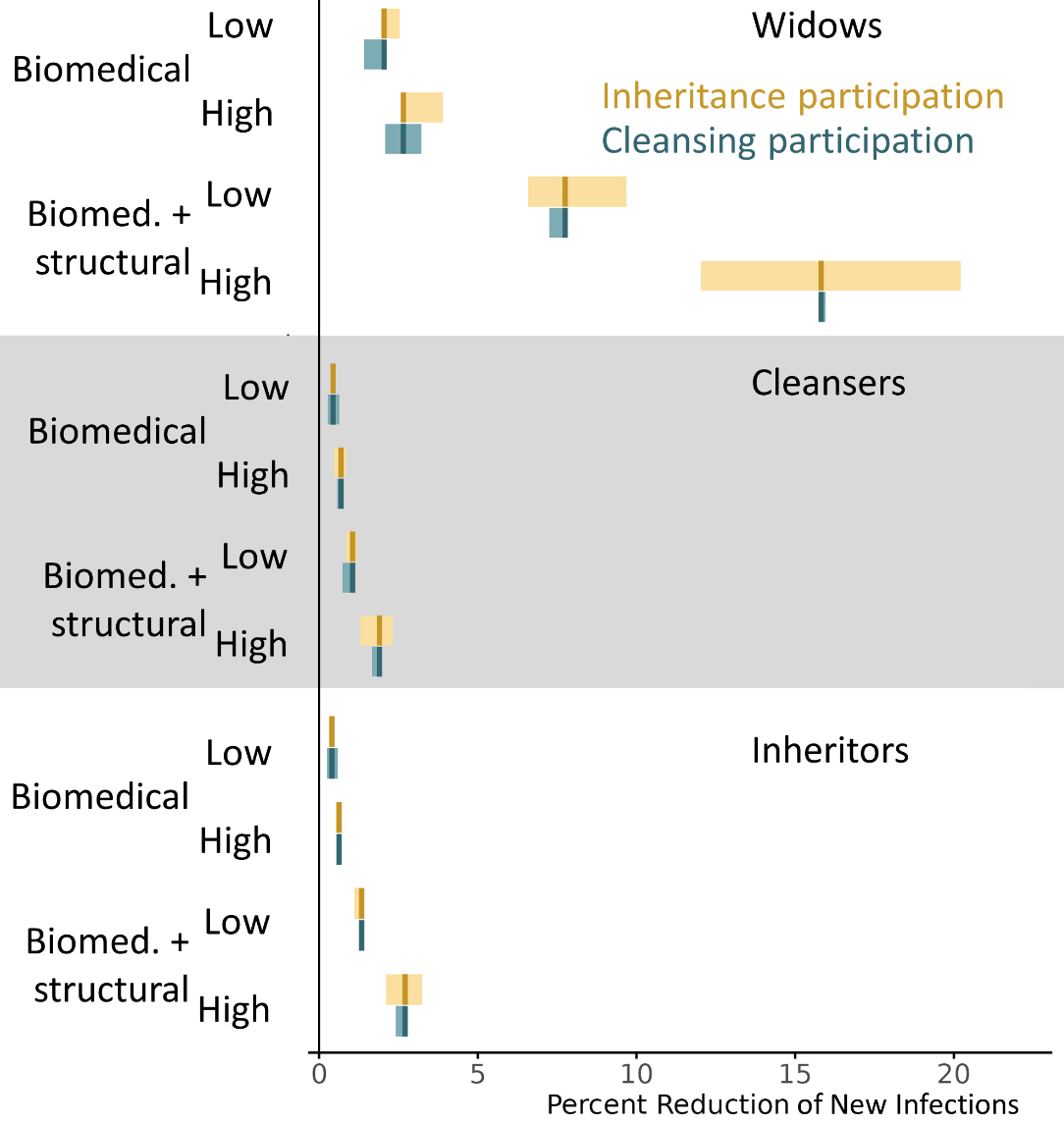


**Figure 5: Percent reduction in new HIV infections when biomedical and structural interventions are provided, and sensitivity to assumed inheritance and cleansing participation rates.** Biomedical interventions included universal HIV testing for widowed women, cleansers, and inheritors, with HIV treatment initiation for those tested positive. For those tested HIV-negative, biomedical interventions included either low (30%) or high (70%) uptake of HIV pre-exposure prophylaxis (PrEP) with 95% efficacy. For inherited widows and their inheritors, PrEP was assumed to continue for one year after the inheritance occurs. Structural interventions were assumed to reduce the exposure of widowed women to widow cleansing and wife inheritance by 30% or 70% (low vs. high uptake). All scenarios are compared to a scenario without biomedical or structural interventions. The range of percent reduction represents the sensitivity to varying widow participation rate in the inheritance (gold) and cleansing (blue) practices. Inheritance participation rate was varied from 41.4% to 71.4%. Cleansing participation rate was varied from 25.6% to 55.6%.
